# Improving Agar Plate Image Quality Using a Low‐Cost 3D‐Printed Lightbox: A Proof‐of‐Concept Evaluation for Machine Learning–Assisted Analysis

**DOI:** 10.1002/mbo3.70399

**Published:** 2026-09-21

**Authors:** Liam A. O'Callaghan, Matthew Olsen, Caleb Kam, Matthew Linnanne, Adrian Goldsworthy

**Affiliations:** ^1^ Faculty of Health Sciences and Medicine Bond University Gold Coast Queensland Australia; ^2^ School of Allied Health, Sport and Social Work Griffith University Nathan Queensland Australia; ^3^ Physiotherapy Department The Prince Charles Hospital Chermside Queensland Australia; ^4^ Critical Care Research Group The Prince Charles Hospital Chermside Queensland Australia; ^5^ Institute for Molecular Bioscience The University of Queensland Brisbane Queensland Australia

**Keywords:** 3D printing, agar plate imaging, colony counting, machine learning, microbiology, open‐source hardware

## Abstract

Agar plate photography is widely used to document microbial growth, but images captured under routine laboratory lighting are often affected by glare, reflections, shadows, and uneven illumination, which may compromise both visual interpretation and computational analysis. This proof‐of‐concept study developed and evaluated a low‐cost, three‐dimensional (3D)‐printed lightbox for standardized smartphone‐based agar plate imaging. Image characteristics were assessed across five uncultured agar media under traditional and lightbox conditions using Fiji/ImageJ. Machine learning–assisted analysis was then performed on cultured horse blood agar plates using QuPath. Separate Random Trees pixel classifiers were developed for traditional and lightbox images to distinguish colonies from background agar and noncolony artifacts. A semiautomated QuPath–assisted workflow was also compared with manual colony counts across 14 growth‐positive horse blood agar plates. Lightbox imaging reduced reflective artifact, improved illumination consistency, and enhanced visualization of colony margins and hemolysis‐associated changes. Traditional‐lighting images produced more artifact‐related classifier detections, including glare, reflections, and uncolonized agar being detected as colony‐like regions. In the exploratory colony‐count comparison, semiautomated QuPath–assisted counts showed close agreement with manual reference counts (Pearson *r* = 0.999; mean absolute error = 2.4 colonies per plate). These findings demonstrate that a low‐cost, 3D‐printed lightbox can improve the quality and standardization of smartphone‐based agar plate images and support more interpretable machine learning–assisted analysis. Further validation using larger independent data sets, additional organisms, media types, and imaging devices is required.

## Graphical abstract

A low‐cost, three‐dimensional‐printed lightbox improved smartphone‐based agar plate imaging by reducing artifacts and enhancing feature visibility. Standardized lightbox images also supported more interpretable QuPath–assisted colony analysis, with exploratory semiautomated counts showing close agreement with manual reference counts.

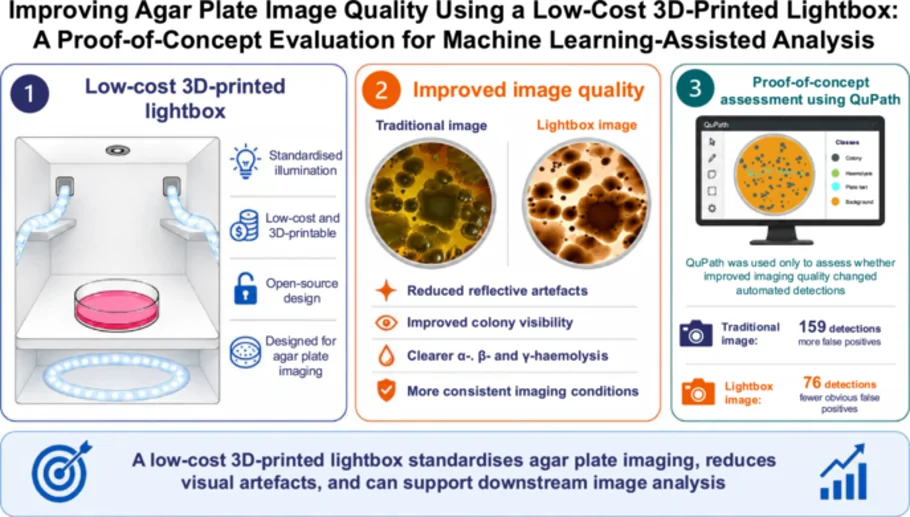

## 1 Introduction

Agar plate imaging is a routine yet increasingly important component of microbiology, supporting the documentation of colony growth, morphology, pigmentation, hemolysis, and other phenotypic features (Rhoads et al. 2015). Quantification of colony‐forming units (CFUs) remains one of the most widely used approaches for estimating viable microbial load, yet manual CFU counting is consistently described as labor intensive, time consuming, and subject to human error (Brugger et al. 2012; Zhu et al. 2018). Machine learning software provides opportunities for rapid and accurate analysis of cultured agar plate images, enabling automated CFU counting, morphological comparison, and quantitative assessment of media color changes indicative of specific metabolic processes, such as pH shifts or hemolysis (Signoroni et al. 2023; Jacot et al. 2024). These approaches depend on consistent, high‐quality images, as the accuracy and reliability of downstream interpretation are determined by the quality of the image acquired at the point of capture. Although commercial automated colony counters provide integrated imaging and analysis, their high cost limits their accessibility for teaching laboratories, smaller research groups, and other resource‐limited settings (Interscience 2026; schuett‐biotec GmbH 2026; Synoptics L 2026).

Obtaining high‐quality images of agar plates is challenging. Petri dishes and agar surfaces differ in their reflective and optical properties, and images captured under routine room lighting or using handheld mobile phones are often affected by glare, reflections, shadows, and uneven illumination (Brugger et al. 2012; K. P. Smith and Kirby 2020). These artifacts substantially alter pixel intensity distributions across agar plate images, introducing nonbiological variation or noise which presents challenges to automated analysis (P. Smith and Schuster 2021). For hemolysis assessment, the illumination strategy is especially important. The American Society for Microbiology recommends that hemolytic reactions on blood agar should be assessed with the light source positioned behind the agar plate, to enhance visualization of zones surrounding colonies that may otherwise be faint or difficult to delineate (Buxton 2016). Savardi et al. (2018) developed an automated hemolysis detection system using top‐ and bottom‐lit images. Their system exploited the complementary information provided by different illumination modes to extract image features and support classification, demonstrating that illumination strategy is central to reliable computational discrimination of α‐, β‐, and γ‐hemolysis (Savardi et al. 2018).

P. Smith and Schuster (2021) recently described an inexpensive imaging box for microbial growth on agar plates, showing that controlled diffuse illumination can greatly reduce lighting artifact compared with conventional bench‐top imaging. Their work highlighted the educational value of high‐quality plate imaging demonstrating that low‐cost solutions are useful in teaching and research settings (P. Smith and Schuster 2021). However, their apparatus was assembled from porous materials (cardboard, wood, and fabric), preventing its reuse, and the aperture was tailored to a specific camera. Importantly, they noted that use with other cameras or mobile phones could introduce light pollution and suggested that future iterations incorporate three‐dimensional (3D)‐printed adapters to improve generalizability and performance (P. Smith and Schuster 2021).

While commercially available photography and machine learning–based culture plate analysis systems exist, their high cost may limit accessibility for many researchers, clinicians, and educational institutions. This highlights the need for low‐cost, effective, and standardized imaging solutions (Signoroni et al. 2023). Open‐source hardware, 3D printing plans, and machine learning software provide opportunities to fabricate low‐cost and customizable laboratory equipment that support machine learning microbiology workflows (Baden et al. 2015). This approach is particularly attractive in educational contexts, where students benefit not only from learning how to take professional‐quality images but also from exposure to machine learning analysis models and workflows. Freely available machine learning platforms such as QuPath support this approach by providing opportunities to students to rapidly develop machine learning models without requiring coding expertise or advanced technical skills, instead focusing on their understanding of microbiology and ability to correctly recognize specific features on agar plates (Signoroni et al. 2023).

The primary aim of this study was to develop a low‐cost, 3D‐printed lightbox for standardized smartphone‐based agar plate photography. Specifically, the study examined whether controlled top and bottom illumination could reduce reflections, shadows, and uneven illumination while improving the visualization of colony‐ and media‐associated features. As a secondary exploratory aim, QuPath pixel classification was used to examine whether images captured with the lightbox were more suitable for machine learning–assisted analysis than those captured under routine laboratory lighting.

## 2 Methods

### 2.1 Study Design

This proof‐of‐concept study was conducted in three stages: (1) design and fabrication of a low‐cost 3D‐printed lightbox for standardized agar plate imaging; (2) assessment of image quality and pixel color distribution in uncultured agar plates imaged under traditional and lightbox conditions; (3) exploratory evaluation of image suitability for trainable QuPath pixel classification using cultured horse blood agar plates.

### 2.2 Lightbox Design

**Figure 1:**
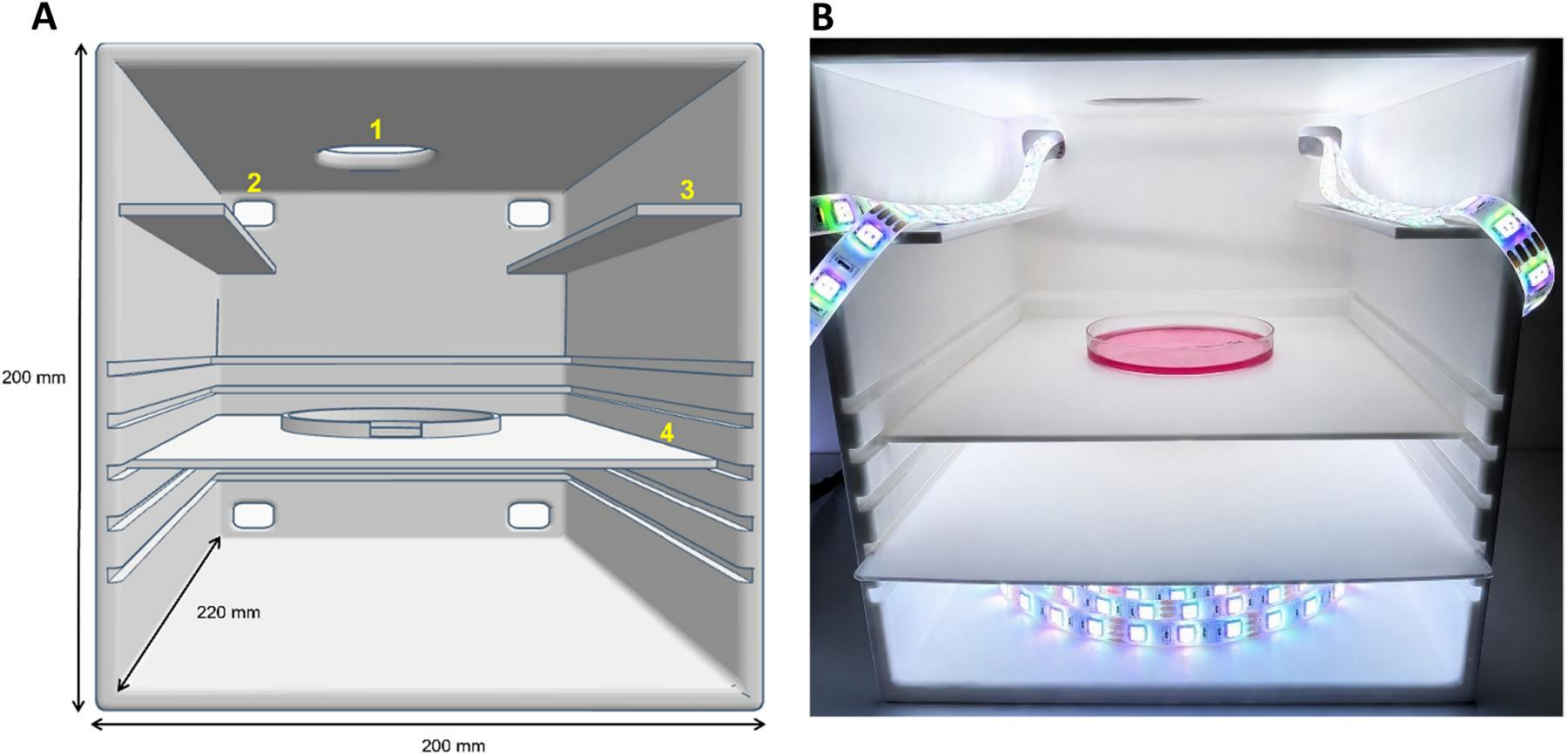
Design and physical prototype of the 3D‐printed agar plate imaging lightbox. (A) Rendered internal view of the lightbox design, showing the main structural features used to standardize agar plate photography. Labeled features include as follows: 1, camera aperture; 2, LED wiring access holes; 3, upper support stage for top‐mounted LEDs; 4, height‐adjustable agar plate holder. The internal chamber dimension is approximately 220 mm. (B) Assembled 3D‐printed prototype showing the LED strip arrangement and agar plate positioning used for image capture. 3D, three‐dimensional; LED, light‐emitting diode.

A custom lightbox was designed to enable consistent smartphone‐based photography of agar plates under controlled illumination (Figure 1). The initial model was developed in Tinkercad and subsequently refined in Autodesk Fusion. The device incorporated an enclosed imaging chamber, an internal platform for agar plate positioning, removable diffusion and light‐blocking components, and integrated light‐emitting diode (LED) lighting to minimize glare, reflection, and uneven illumination during image capture. The lightbox was designed to accommodate standard 90 mm microbiology Petri dishes. A modular plate‐support insert enabled the plate to be positioned centrally within the imaging chamber and could be modified to accommodate alternative plate sizes. An aperture at the top of the device enabled reproducible positioning of a smartphone camera directly above the agar plate, thereby standardizing imaging distance, camera angle, and plate position between photographs. Illumination was provided by two independently configurable LED strips positioned above and below the agar plate. The lower light source provided transmitted illumination through the agar, while the upper light source provided reflected illumination to assist visualization of colony morphology, pigmentation, and media‐associated changes. A removable 1‐mm diffuser plate, printed in white, was positioned above the lower light source to reduce the intensity of direct LED illumination and improve lighting uniformity. The diffuser is intended for use when a bright dedicated light source, such as the LED strip, is used beneath the Petri dish. A removable blocker plate, printed in black, was used to isolate individual illumination configurations.

### 2.3 Fabrication and Recommended Printing Parameters

The lightbox was fabricated using an ELEGOO Centauri Carbon fused deposition modeling printer with a build volume of 256 mm × 256 mm × 256 mm and a standard 0.4‐mm nozzle. White polylactic acid (PLA) filament was used for all printed components of the prototype. PLA was selected because it is inexpensive, widely available, and compatible with most desktop 3D printers. White filament was used to enhance internal light reflection and promote more uniform illumination, thereby reducing shadows. The main lightbox enclosure measured approximately 220.7 mm depth × 200 mm height × 200 mm width. Additional printed components included an agar plate‐support insert measuring 191 mm × 200 mm × 2.5 mm with an approximately 85.7‐mm circular aperture, a diffuser plate (printed in white) measuring 191 mm × 230 mm × 1.0 mm, and a blocker plate (printed in black) measuring 191 mm × 230 mm × 2.5 mm. Depending on print settings, the main enclosure required approximately 700–900 g of filament, corresponding to an estimated material cost of approximately AU$14–36.

Recommended print settings were a layer height of 0.20 mm, at least three wall perimeters and 15% adaptive cubic or gyroid infill. Print speed was set according to the default settings appropriate for the printer and filament. Supports were not required when components were printed in the recommended orientation. A brim was recommended for the main enclosure, particularly when using a nonenclosed printer, but was not generally required for the smaller components. The main enclosure should be printed in the backwards‐rotated orientation provided in the accompanying 3MF project file to minimize overhangs and avoid the need for support material. Illumination was provided by two low‐cost ANKO 3‐m LED light strips, each costing approximately AU$15. The lightbox design files, assembly notes and supporting workflow materials are freely available through an open‐access repository (Github.com) to support reuse, modification, and local fabrication (https://github.com/drliamocallaghan/agar-plate-lightbox.git).

### 2.4 Assessment of Pixel Intensity Distribution in Uncultured Plates

The performance of the lightbox was evaluated using uncultured agar plates (bile aesculin, nutrient, horse blood, mannitol salt, and MacConkey agar). Photographs were captured under three imaging conditions, including a traditional method in which agar plates were placed on a black background, imaging within the lightbox using top lighting only, and imaging within the lightbox using combined top and bottom lighting. All photographs were captured using the same iPhone 13 Pro (iOS version 18.5) with identical camera settings. Flash was disabled for all images, and no exposure adjustment was applied. Plates were positioned centrally within the imaging field, and all photographs were taken from a fixed distance using the standardized aperture of the lightbox. Captured images were analyzed using Fiji/ImageJ version 2.16.0/1.54 s. Each image was cropped to include the agar area within the bottom rim of the plate. Glare and illumination characteristics were assessed using 3D surface plots and pixel color histograms. In the surface plots, pixel color was represented as elevation, such that reflective artifacts appeared as sharp peaks. Histograms were used to examine the distribution of pixel color, with a greater dispersion of pixel colors evidence of uneven illumination.

### 2.5 Development and Testing of Machine Learning Models on Cultured Horse Blood Agar

Twenty horse blood agar plates were prepared as part of a broader mobile‐phone sampling study. Samples were collected from mobile phones of hospital staff within an intensive care unit environment using sterile flocked swabs containing 1 mL of Liquid Amies transport medium (COPAN ESwab). An additional 1 mL of Liquid Amies was added to each sample suspension before vortexing for 5 s, after which 100 µL was spread onto each horse blood agar plate using a sterile L‐spreader. Plates were incubated at 37°C for 48 h in 5% CO_2_. Following incubation, photographs were captured under traditional and lightbox imaging conditions using the same smartphone and camera settings described in Section 2.4. Fourteen plates demonstrated microbial growth and were included in the exploratory QuPath analysis. The remaining six plates corresponded to samples collected following a phone‐cleaning intervention undertaken as part of the broader study and showed no microbial growth. As no colonies were present for classification or counting, these plates were not included in the QuPath analysis. Analysis was restricted to horse blood agar as a proof‐of‐concept, as evaluation of additional media would require development and optimization of separate classifiers because of differences in media color and image characteristics.

**Figure 2:**
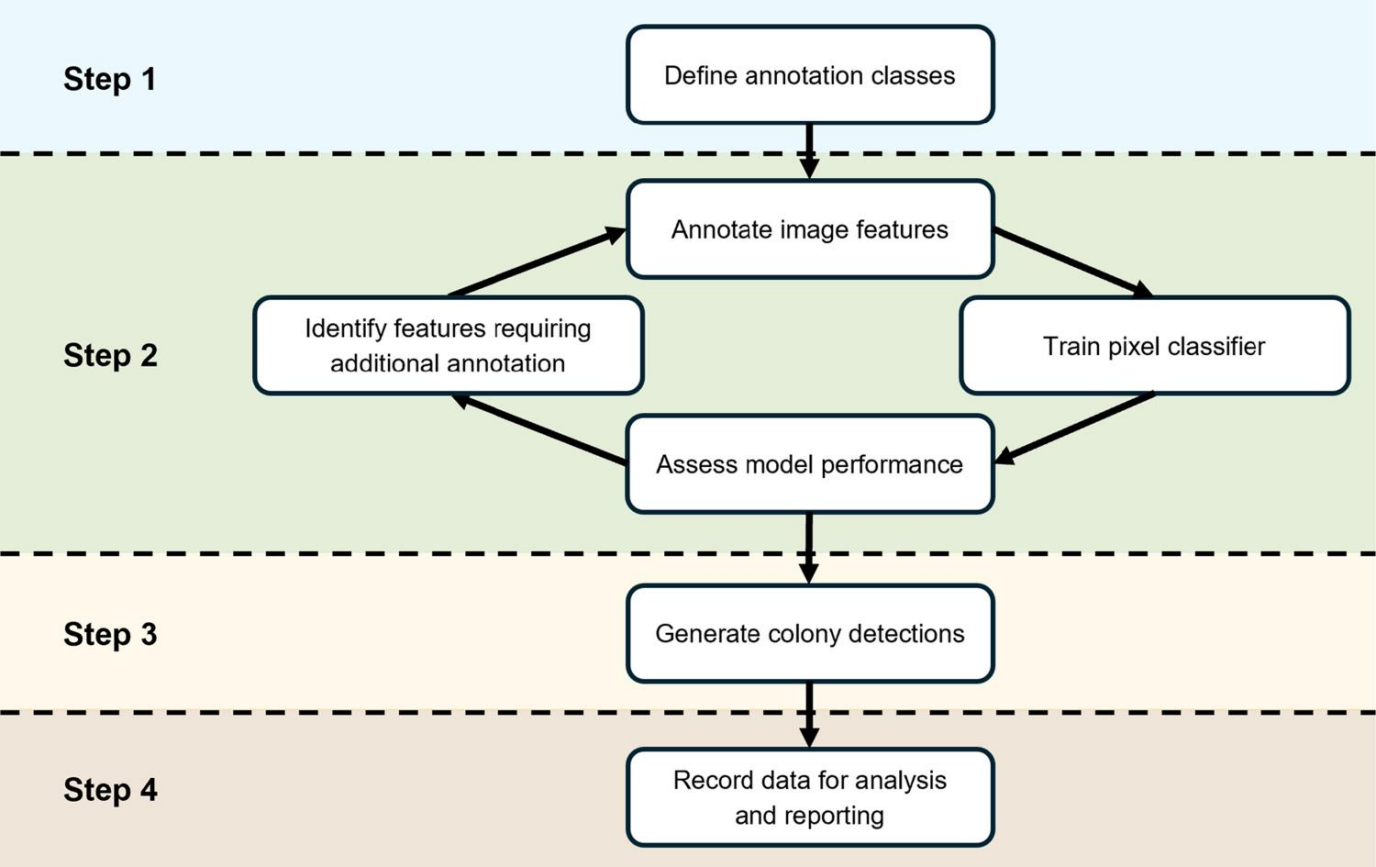
Exploratory QuPath pixel‐classification workflow used to assess agar plate image suitability. Step 1: Annotation classes were defined for bacterial colonies, background agar, and noncolony artifacts, including printed plate text and reflective regions. Step 2: Representative image regions were manually annotated, and separate Random Trees pixel classifiers were trained for the traditional and lightbox imaging conditions. Classification overlays were visually reviewed, and annotations were iteratively refined. Step 3: Pixels classified as colonies were converted into detection objects. Step 4: Colony detection outputs were recorded for analysis and reporting.

Exploratory machine learning–assisted image analysis was performed using QuPath version 0.7.0 (Bankhead et al. 2017). Manual training annotations were created by three authors (LO, AG, and MO) for three classes: bacterial colonies, background agar, and noncolony artifacts. The noncolony artifact class included printed plate text, reflective regions, and other image features that could visually resemble colonies. Separate Random Trees pixel classifiers were developed for traditional and lightbox images of the same plates, allowing the effect of image‐acquisition conditions to be compared while holding the underlying colony distribution constant. Pixel classification was performed at full image resolution using an iterative training process (Figure 2). Classification overlays were visually reviewed, and additional annotations were added to incorrectly classified regions to improve separation between classes.

### 2.6 Descriptive Evaluation of QuPath Classifier Outputs

To support comparison between imaging conditions, the traditional and lightbox classifiers received the same number of training annotations. Classifier outputs were evaluated descriptively by visually comparing detections with the corresponding agar plate images. Particular attention was given to artifact‐related detections, missed colonies, and fragmentation of individual colonies into multiple detections. The total number of detections generated under each imaging condition was also recorded. Because classifier refinement and descriptive evaluation were performed using the same images, no separate validation or holdout test data set was used. Classifier outputs were therefore interpreted as exploratory comparisons of behavior under the two imaging conditions rather than formal estimates of model accuracy or generalizability. A separate exploratory comparison of semiautomated QuPath–assisted and manual colony counts is described in Section 2.7.

### 2.7 Exploratory Comparison With Manual Colony Counts

To further explore the utility of the QuPath‐assisted workflow, colony counts were compared with manual reference counts across the 14 growth‐positive horse blood agar plates included in the exploratory analysis. A manual colony count was performed for each image and treated as the reference count. For the semiautomated workflow, lightbox images were initially analyzed using the QuPath classifier utilizing an artificial neural network, after which the resulting detections were visually reviewed by a manual assessor and adjusted where required. The relationship between semiautomated and manual counts was assessed using Pearson's correlation coefficient (*r*), while absolute counting error was assessed using mean absolute error (MAE). These analyses were considered exploratory and were not intended as independent validation of a fully automated colony‐counting system.

## 3 Results

### 3.1 Assessment of Pixel Color Distribution in Uncultured Plates

Compared with traditional photography, imaging within the lightbox produced a more even pixel color distribution across all agar plate conditions (Table 1). Photographs captured under standard lighting conditions showed greater reflective artifacts, including visible reflections from the iPhone and the biosafety cabinet filter screen, which contributed to increased dispersion of pixel color across the agar surface. In contrast, lightbox images showed reduced pixel color dispersion and more uniform color distribution across the plate. Some variation was observed between agar types and lighting configurations. More transparent agar plates tended to display a slight color gradient when imaged using combined top and bottom lighting. Factory‐printed text on the underside of the agar plates was visible under all imaging conditions; however, the contrast between the agar and printed text was more pronounced in the combined top and bottom lighting condition. The rim of the agar plate introduced additional edge‐related noise across all lighting conditions, but this was easily removed by cropping the images to the agar area within the bottom rim of the plate using ImageJ.

**Table 1:** Pixel color dispersion of uncultured agar plates under standard, top‐lit, and combined top‐and‐bottom‐lit conditions.

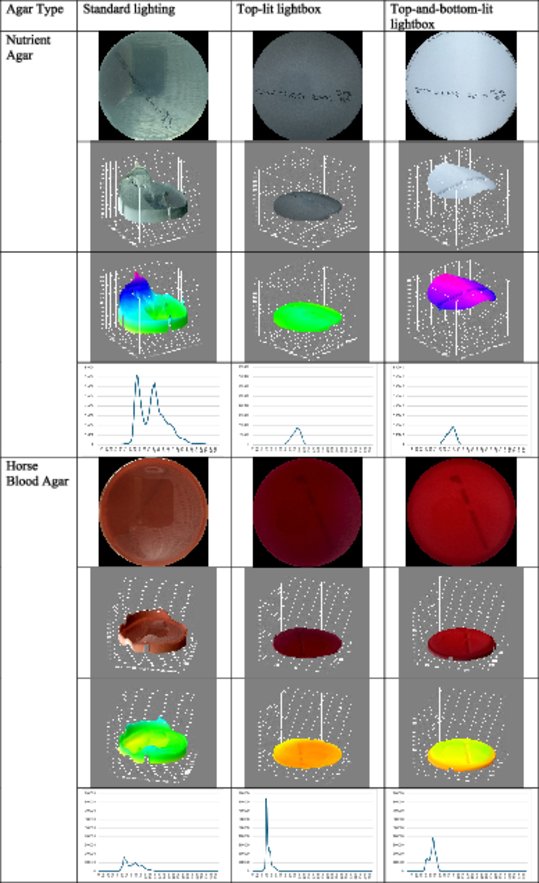
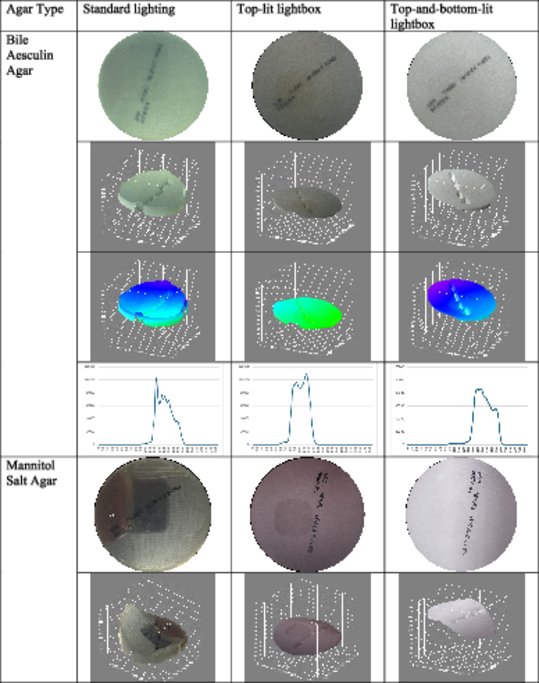
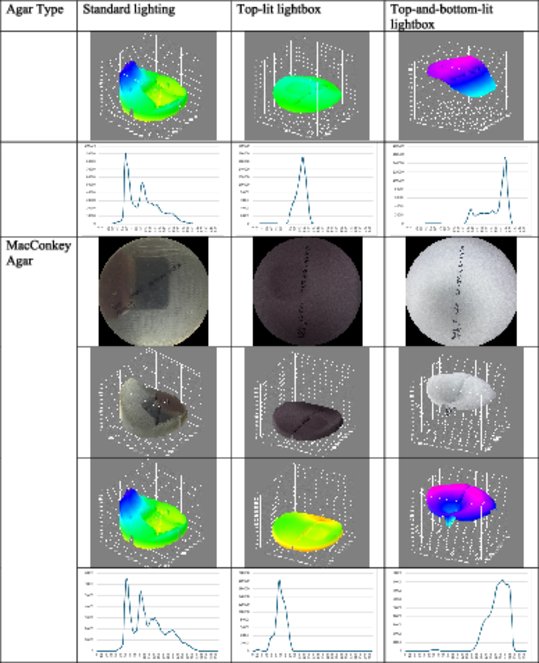

### 3.2 Assessment of Photograph Quality of Cultured Plates

**Figure 3:**
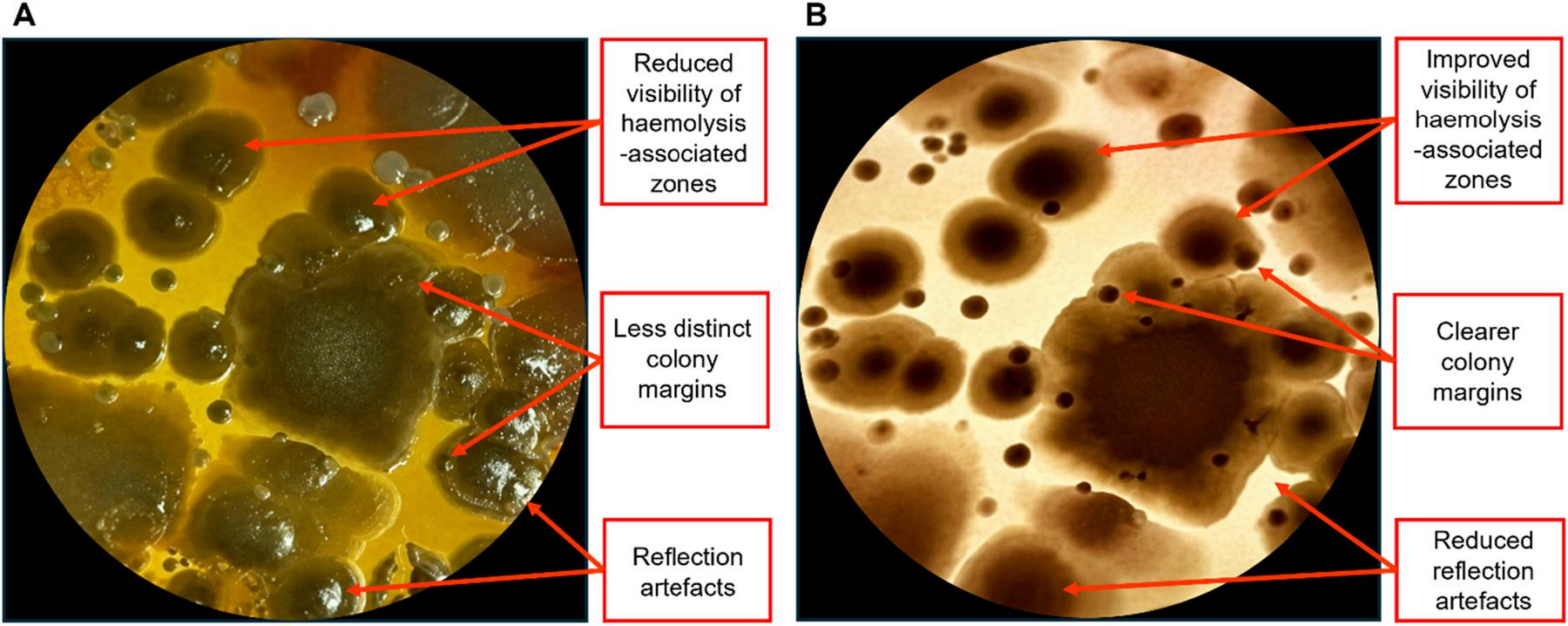
Improved visualization of colony and hemolysis‐associated features using lightbox imaging. (A) Representative horse blood agar image captured under traditional‐lighting conditions, showing reflective artifact, variable contrast, and less distinct visualization of colony‐associated media changes. (B) Corresponding image captured using the lightbox under combined top and bottom lighting, showing reduced reflective artifact, clearer colony margins, and improved visualization of hemolysis‐associated color changes.

Photographs captured using the traditional‐lighting approach commonly displayed reflective artifacts across uncolonized regions of the agar surface, as well as small regions of reflection on individual colonies, irrespective of colony morphology or hemolytic pattern (Figure 3). In contrast, images captured using the lightbox showed reduced reflective artifact and improved visualization of colony margins. The combined top‐ and bottom‐lighting condition produced greater color and contrast differentiation between regions of α‐, β‐, and γ‐hemolysis, improving the visual distinction between hemolytic patterns. Transmitted illumination through the agar also improved the visibility of small or low‐contrast colonies that were less apparent under traditional imaging conditions.

### 3.3 Comparison of QuPath Machine Learning Detection Outputs

**Figure 4:**
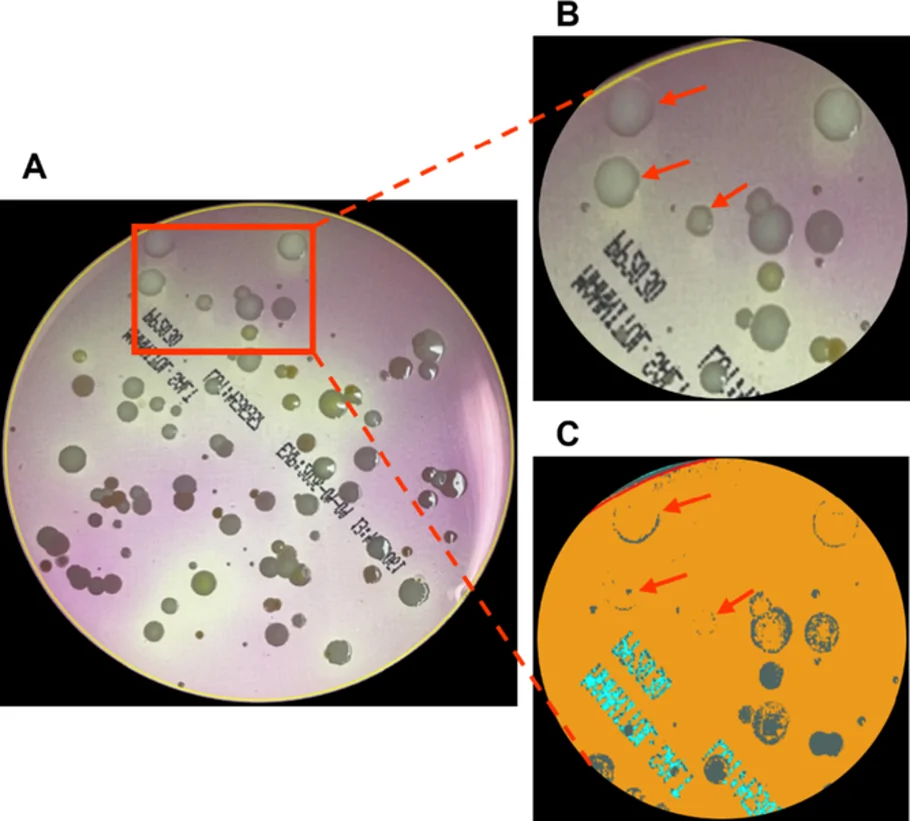
Inconsistent colony detection by the machine learning model under traditional‐lighting conditions. (A) Representative agar plate image captured under traditional lighting, with the selected region of interest indicated by the red box. (B) Magnified raw image of the selected region, showing colonies with variable contrast near printed plate text. (C) Corresponding QuPath classifier output, showing incomplete and inconsistent colony detection within the same region. Red arrows indicate colonies visible in the raw image and corresponding areas that were missed, poorly segmented, or inconsistently classified in the model output. These errors were most apparent in regions affected by uneven illumination, printed plate text, and reduced contrast between colonies and background agar.

**Figure 5:**
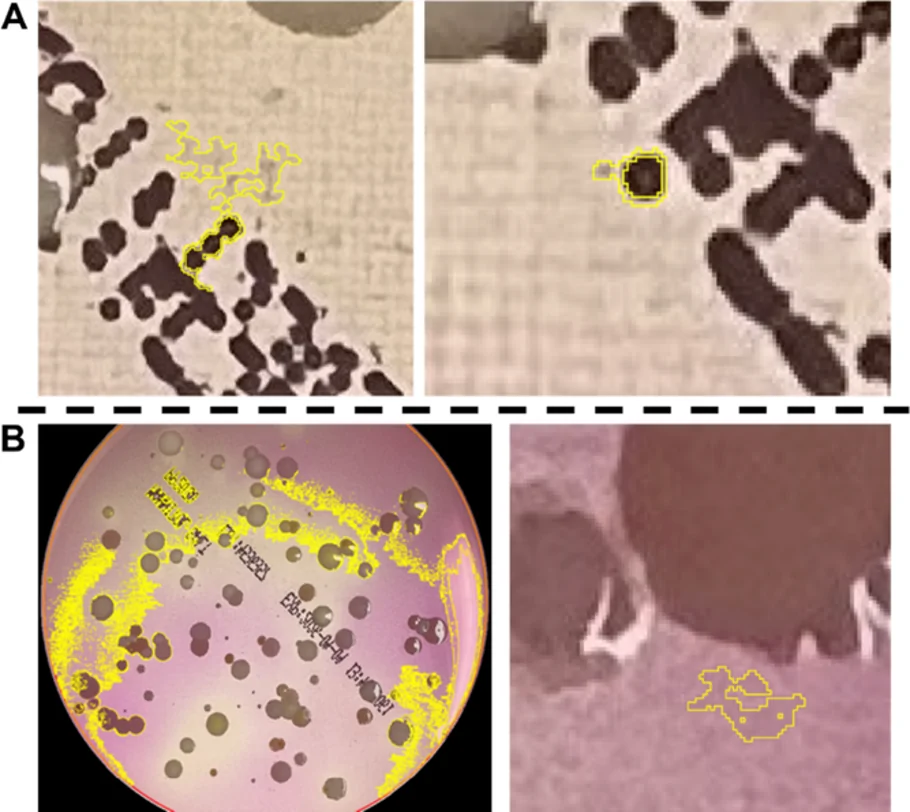
Representative false‐positive detections produced by the machine learning model under traditional‐lighting conditions. Yellow outlines indicate regions classified as colonies by the QuPath model. (A) Printed text on the underside of the agar plate was incorrectly detected as colony‐like objects. (B) Reflective artifacts and unevenly illuminated regions of uncolonized agar were incorrectly classified as colonies. These examples demonstrate how nonstandardized lighting and background artifacts can inflate colony detections and reduce the interpretability of machine learning–assisted colony analysis.

**Figure 6:**
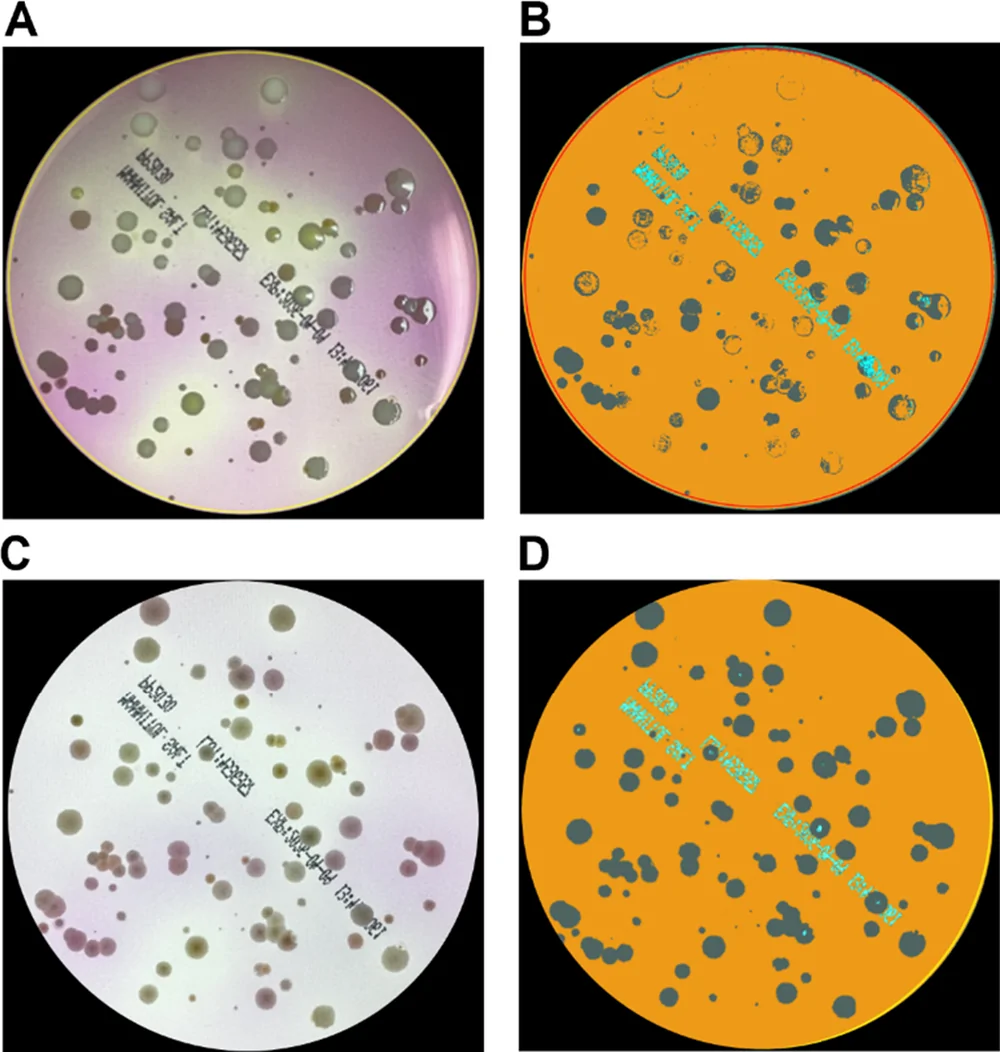
Representative comparison of machine learning–assisted colony detection under traditional and lightbox imaging conditions. (A) Agar plate image captured under traditional‐lighting conditions. (B) Corresponding QuPath classifier output generated from the traditional‐lighting image, showing increased false‐positive detections, including the classification of printed plate text and artifact as colony‐like regions. (C) Agar plate image captured using the lightbox under combined top and bottom lighting. (D) Corresponding QuPath classifier output generated from the lightbox image, showing more consistent colony detection and reduced artifact‐related misclassification.

For the representative matched plate shown in Figures 4, 5, 6, the classifier trained on the traditional‐lighting image generated 159 detections, with visual review identifying several detections associated with noncolony features. These included printed plate text being detected as colony‐like objects (Figure 5A), uncolonized agar regions being classified as colonies (Figure 5B), and fragmentation of individual colonies into multiple detections. In comparison, the classifier trained on the corresponding lightbox image generated 76 detections and showed fewer visually apparent artifact‐related detections following review (Figure 6). These values represent outputs from a single representative plate pair and were used to illustrate differences in classifier behavior between imaging conditions rather than as aggregate measures of model performance. Overall, the findings suggest that lightbox imaging improved the interpretability of classifier outputs by reducing interference from acquisition‐related artifacts. An exploratory comparison of semiautomated QuPath–assisted counts with manual reference counts across the 14 growth‐positive plates is presented in Section 3.4.

### 3.4 Exploratory Comparison With Manual Colony Counts

**Figure 7:**
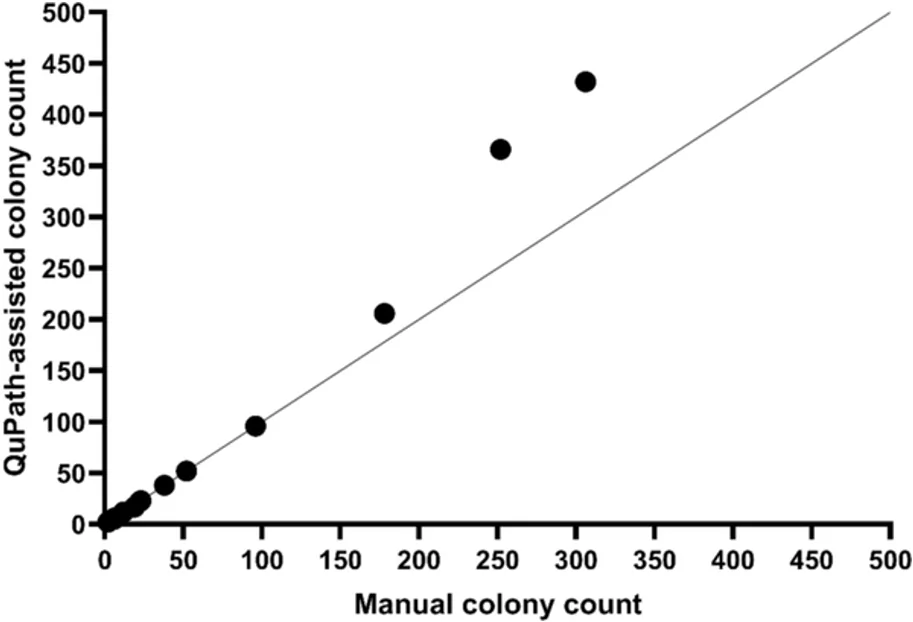
Exploratory comparison of manual and QuPath‐assisted colony counts. Each point represents one growth‐positive horse blood agar plate (*n* = 14). The solid line represents perfect agreement (*y* = *x*). Colony counts obtained using the semiautomated QuPath–assisted workflow showed close agreement with manual reference counts.

Across the 14 growth‐positive horse blood agar plates, manual assessment identified 1014 colonies, compared with 992 colonies using the semiautomated QuPath–assisted workflow. The semiautomated and manual counts showed a strong linear association (Pearson *r* = 0.999), with an MAE of 2.4 colonies per plate (Figure 7). These exploratory findings indicate close agreement between manual counting and the QuPath‐assisted workflow following manual review, although larger independent data sets are required to establish its generalizability.

## 4 Discussion

This proof‐of‐concept study developed a low‐cost, 3D‐printed lightbox for standardized smartphone‐based imaging of agar plates. Compared with routine laboratory photography, the lightbox reduced reflections and uneven illumination and improved the visualization of colony margins and hemolysis‐associated changes. Exploratory QuPath analysis further demonstrated how image‐acquisition artifacts, including reflections, printed plate text, and unevenly illuminated agar, can interfere with trainable pixel classification. The quality and consistency of image acquisition are increasingly important as microbiology laboratories adopt digital documentation and computational image analysis. Images captured under uncontrolled conditions may contain substantial nonbiological variation arising from differences in illumination, imaging distance, camera angle, and background reflection. By standardizing plate position, camera position, and illumination, the lightbox provides a reproducible approach to reducing these sources of variation using equipment that can be fabricated with commonly available desktop 3D printers.

### 4.1 Comparison With Existing Imaging Approaches

The findings are consistent with previous work demonstrating that controlled illumination can improve agar plate photography. P. Smith and Schuster (2021) described a low‐cost imaging apparatus constructed from cardboard, wood, and fabric that reduced lighting artifacts compared with conventional photography. The present design builds upon this approach through reusable 3D‐printed components, a fixed camera aperture, modular plate positioning, and independently configurable top and bottom illumination. These features support consistent local fabrication and allow the design to be adapted for different plate sizes, imaging devices, and laboratory requirements. Gerada et al. also incorporated controlled plate imaging using a commercially available lightbox into a machine learning–assisted agar‐dilution workflow (Gerada et al. 2024). Their study demonstrates how standardized image acquisition can contribute to a formally validated analytical system. In contrast, the present study focuses primarily on the development of an accessible and reproducible image‐acquisition device for use prior to either human interpretation or computational analysis.

Commercial plate‐imaging and automated colony‐counting systems may cost more than USD $20,000, limiting their accessibility for teaching laboratories, smaller research groups, and resource‐limited settings. The open release of the present design files provides a substantially lower‐cost alternative and supports reproducibility by allowing laboratories to fabricate, modify, and independently validate the imaging system using accessible desktop 3D‐printing workflows.

### 4.2 Standardized Image Acquisition for Machine Learning Analysis

The exploratory QuPath findings illustrate how nonbiological image features can influence classifier outputs. Under routine lighting, reflections on colonies and uncolonized agar sometimes displayed similar pixel characteristics, making it difficult for the classifier to distinguish genuine colonies from imaging artifacts. Printed text on the underside of agar plates also remained problematic, particularly when its color and contrast resembled colonies or hemolysis‐associated changes. Images captured using the lightbox produced fewer visually apparent detections associated with reflections, printed text, and uneven illumination. These observations align with broader principles of data quality in machine learning. The METRIC framework for trustworthy medical artificial intelligence highlights consistency and representativeness as key dimensions of training‐data quality (Schwabe et al. 2024). Variation in imaging conditions between training and test data sets can also contribute to domain shift and reduce model generalizability (Kilim et al. 2022). Standardizing illumination, imaging distance, camera angle, and plate position may therefore help reduce acquisition‐related variability and improve data set consistency.

However, these findings should not be interpreted as evidence of validated colony‐counting accuracy, as the classifiers were developed and evaluated using the same image set without an independent holdout data set. The QuPath workflow, therefore, primarily demonstrates improved image suitability for analysis rather than validated model performance. An exploratory comparison with manual colony counts provided further support for the potential utility of the QuPath‐assisted workflow. Across 14 horse blood agar plate images, semiautomated counts following manual review showed close agreement with manual reference counts (Pearson *r* = 0.999; MAE = 2.4 colonies per plate, Figure 7). These findings suggest that QuPath‐assisted colony assessment may be useful when combined with human oversight, although larger independent data sets are required before the workflow can be considered validated. Additional preprocessing steps, such as plate masking, rim exclusion, and explicit annotation of printed text, may further improve robustness.

Open‐source tools such as OpenCFU and AutoCellSeg have previously been used for automated colony counting, particularly in images with discrete and well‐separated colonies (Khan et al. 2018; Geissmann 2013). However, agar plate images frequently include confluent growth, irregular morphology, media color variation, and reflective artifacts that complicate segmentation. QuPath offers flexible annotation, pixel classification, and scripting tools that may better accommodate these complexities (Bankhead et al. 2017). Nevertheless, larger annotated data sets and formal validation across organisms, media types, and imaging conditions would be required before generalizable automated workflows can be established.

### 4.3 Standardized Image Acquisition in Education and Training

Beyond computational applications, the lightbox may also have value in microbiology education. Agar plate interpretation requires recognition of colony morphology, pigmentation, hemolysis, and media‐associated changes, but inconsistent imaging can make these features difficult to demonstrate reliably across teaching materials. Standardized photography could therefore support the creation of more consistent image sets for practical teaching, assessment, and online learning resources. It may also provide a useful platform for introducing students to digital image analysis and machine learning concepts. By comparing images captured under controlled and uncontrolled conditions, students can identify imaging artifacts and explore how data quality and annotation influence computational outputs. This aligns with current microbiology curriculum priorities, including experimental design, troubleshooting, quantitative reasoning, and data interpretation (American Society for Microbiology 2024; Boury et al. 2024). Importantly, the educational value of the device does not depend on QuPath functioning as a validated colony‐counting system. Instead, it can be used to demonstrate how image acquisition and data set quality directly affect downstream analysis, a principle relevant across microbiology, histopathology, and radiology.

### 4.4 Considerations for Agar Plate Design

Several plate‐related features influenced image analysis. Many manufacturers print information on the underside of agar plates, including agar type, batch details, and expiry information. Although improved lighting increased contrast between colonies and background agar, printed text remained problematic in some conditions, particularly on opaque media such as horse blood agar, where it appeared as darker, poorly defined regions that could resemble colonies or hemolytic changes. Rather than requiring complete exclusion of these regions, this highlights the need to train image classifiers to recognize printed text as a separate image class distinct from colonies and background agar. However, this increases the annotation burden and may require additional training examples, particularly when text is partially obscured or visually similar to darker colonies or hemolytic regions. Similarly, the circular rim on the base of agar plates introduced edge‐related artifact, likely due to light refraction and changes in plate geometry.

### 4.5 Limitations and Future Directions

This study should be interpreted as a proof‐of‐concept evaluation rather than a full validation of an automated colony‐counting platform. The number of plates, agar types, microbial morphologies, and imaging devices assessed was limited, and all images were captured using a single smartphone model and one lightbox prototype. As such, device performance may vary with different cameras, exposure settings, LED color temperatures, diffusion materials, agar manufacturers, plate geometries, and 3D‐printing materials. The exploratory QuPath analysis was restricted to horse blood agar, and differences in color and optical characteristics between media would require the development and validation of medium‐specific classifiers before the findings could be generalized to other agar types. Although an exploratory comparison across 14 growth‐positive horse blood agar plates demonstrated close agreement between semiautomated QuPath–assisted counts and manual reference counts, this did not represent independent validation of the workflow. Larger independent data sets incorporating a broader range of organisms, colony densities, and imaging conditions are therefore required, together with comparison against established open‐source and commercial colony‐counting systems.

Future workflows should also address plate‐related artifacts that remained problematic in the present study, including printed text on the underside of agar plates and edge‐related artifacts from the circular plate rim. These issues may be managed through automated plate masking, rim exclusion, and text management before classifier training. The lighting conditions also altered the apparent color and contrast of colonies and agar, which may affect applications in which pigmentation or media color contributes to organism identification. Future studies should therefore assess color fidelity using standardized color references and camera settings. Ambient laboratory light combined with smartphone night‐mode imaging may also provide a simpler alternative to active LED illumination and warrants further evaluation.

Finally, while PLA was suitable for low‐cost prototype development, alternative materials may be preferable for routine laboratory use. Repeated disinfection, particularly with alcohol‐based disinfectants, may place greater demands on material durability, surface cleanability, and chemical resistance. Polyethylene terephthalate glycol or polycyclohexylene dimethylene terephthalate glycol may therefore be more appropriate for long‐term microbiology laboratory use. However, any change in printing material, surface finish, or internal reflectivity should be reevaluated, as these factors may alter illumination conditions and affect the performance of machine learning models trained under specific imaging conditions.

## Author Contributions

**Liam A O'Callaghan:** conceptualization, methodology, investigation, writing – original draft, writing – review and editing, data curation, visualization, formal analysis, project administration. **Matthew Olsen:** writing – original draft, writing – review and editing, resources, investigation, methodology. **Caleb Kam:** resources, investigation, writing – review and editing. **Matthew Linnanne:** investigation, writing – review and editing, resources. **Adrian Goldsworthy:** conceptualization, project administration, formal analysis, data curation, investigation, writing – original draft, writing – review and editing, visualization, methodology.

## Funding

The authors have nothing to report.

## Ethics Statement

The authors have nothing to report.

## Conflicts of Interest

None declared.

## Data Availability

The 3D‐printing design files, workflow materials, and supporting documentation are publicly available in the project repository at https://github.com/drliamocallaghan/agar-plate-lightbox. Additional data supporting the findings of this study are available from the corresponding author upon reasonable request.
